# Detecting Lung Cancer Trends by Leveraging Real-World and Internet-Based Data: Infodemiology Study

**DOI:** 10.2196/16184

**Published:** 2020-03-12

**Authors:** Chenjie Xu, Hongxi Yang, Li Sun, Xinxi Cao, Yabing Hou, Qiliang Cai, Peng Jia, Yaogang Wang

**Affiliations:** 1 School of Public Health Tianjin Medical University Tianjin China; 2 School of Public Health Yale University New Haven, CT United States; 3 School of Nursing Tianjin Medical University Tianjin China; 4 The Second Hospital of Tianjin Medical University Tianjin China; 5 Department of Land Surveying and Geo-Informatics The Hong Kong Polytechnic University Hong Kong China; 6 International Initiative on Spatial Lifecourse Epidemiology Hong Kong China; 7 Faculty of Geo-information Science and Earth Observation University of Twente Enschede Netherlands

**Keywords:** lung cancer, incidence, mortality, internet searches, infodemiology

## Abstract

**Background:**

Internet search data on health-related terms can reflect people’s concerns about their health status in near real time, and hence serve as a supplementary metric of disease characteristics. However, studies using internet search data to monitor and predict chronic diseases at a geographically finer state-level scale are sparse.

**Objective:**

The aim of this study was to explore the associations of internet search volumes for lung cancer with published cancer incidence and mortality data in the United States.

**Methods:**

We used Google relative search volumes, which represent the search frequency of specific search terms in Google. We performed cross-sectional analyses of the original and disease metrics at both national and state levels. A smoothed time series of relative search volumes was created to eliminate the effects of irregular changes on the search frequencies and obtain the long-term trends of search volumes for lung cancer at both the national and state levels. We also performed analyses of decomposed Google relative search volume data and disease metrics at the national and state levels.

**Results:**

The monthly trends of lung cancer-related internet hits were consistent with the trends of reported lung cancer rates at the national level. Ohio had the highest frequency for lung cancer-related search terms. At the state level, the relative search volume was significantly correlated with lung cancer incidence rates in 42 states, with correlation coefficients ranging from 0.58 in Virginia to 0.94 in Oregon. Relative search volume was also significantly correlated with mortality in 47 states, with correlation coefficients ranging from 0.58 in Oklahoma to 0.94 in North Carolina. Both the incidence and mortality rates of lung cancer were correlated with decomposed relative search volumes in all states excluding Vermont.

**Conclusions:**

Internet search behaviors could reflect public awareness of lung cancer. Research on internet search behaviors could be a novel and timely approach to monitor and estimate the prevalence, incidence, and mortality rates of a broader range of cancers and even more health issues.

## Introduction

Cancer affects people at all socioeconomic levels and has become a worldwide public health problem. In 2018, the International Agency for Research on Cancer reported the substantial global burden of cancer [1]. Lung cancer is the most commonly diagnosed cancer type (11.6% of all cases) and is the leading cause of cancer-related deaths (18.4% of all cases). In the United States, cancer is the second leading cause of death, resulting in approximately 150,000 deaths per year [2,3]. Moreover, the mortality rate of lung cancer is the highest in the United States. Current data on cancer incidence, mortality, and survival have been mainly collected by the US Centers for Disease Control and Prevention (CDC) and the National Cancer Institute (NCI). However, the year of disclosing such data usually lags about 3 years behind the year in which the data are relevant owing to the time required for data collection, compilation, quality control, and dissemination [4]. Despite an increasing demand for up-to-date knowledge regarding cancers, the lack of real-time data continues to be a major impediment to timely and effective cancer surveillance [5]. Hence, new methods in the era of big data are needed to help supplement current strategies and improve the monitoring of lung cancer.

Along with the rapid spread of social media and medical forums, people frequently search internet resources for symptom-related information, basic medical advice, and to exchange information [6-10]. As more and more people utilize the internet, data from internet searches are increasingly able to better reflect real-world data. With the advent of big data, information and communication technologies have made it possible to reflect trends of real-world diseases based on search data [11-13].

The past decade has witnessed an exponential increase in internet penetration. As of April 2019, 56% of the world’s population was reported to have internet access, with rates in the developed world now exceeding 80% [14,15]. According to the World Bank, individuals who use the internet accounted for 76% of the total population in the United States in 2017 [16]. Additionally, 95% of Americans now own a cell phone of some kind, and approximately 77% of the US population now owns a smartphone [17]. Digital data passively generated from user online search behaviors may be utilized to estimate disease metrics in different states until verification data from a traditional source becomes available [18,19]. Internet technologies have the potential to mitigate some of the shortcomings of current health monitoring systems and might be used to supplement existing disease surveillance methods.

Previous studies have attempted to utilize search engines (eg, Google and Baidu) to improve the surveillance of some epidemics. Some scholars have made use of online social networks (eg, Facebook and Twitter) to mine public interest in medical-related issues such as diagnosis and treatment knowledge, patient experience sentiments, and quality of medical services. Meanwhile, more and more studies have elucidated trends in disease communications through internet communities (eg, Reddit) [20-22]. Since 2004, Google Trends [23] has provided national- and state-level search data for free via entering related search query terms [23]. Previous studies have revealed that it is possible to improve the surveillance and prediction of infectious diseases and examine public interest in multiple health topics by monitoring the search behaviors of millions of users and conducting data mining through such search engines [24-29]. However, few studies have focused on leveraging internet search data to monitor and predict cancers at a geographically finer state-level scale [30,31].

In the present study, we tested the hypothesis that the volumes of internet search queries related to lung cancer reflect real-world spatiotemporal variation in the incidence and mortality rates of lung cancer in the United States. Our findings suggest that internet search volumes may reflect disease characteristics of lung cancer (such as incidence and mortality) and provide an additional means of national- and state-level cancer surveillance in the United States.

## Methods

### Internet Search Data

We collected monthly search data for lung cancer through Google Trends [23] from 2004 to 2018, at both the state and national levels. The search data were downloaded from Google Trends in January 2019. A research firm reported that Google accounted for up to 92% of the market share of search engines in 2018, representing an increase of 57% and 89% in 2004 and 2015, respectively; thus, Google is currently the most widely used search engine in the world [32].

When downloading search data, we could freely choose our own time range. The earliest search data available for Google Trends are from 2004. Therefore, to illustrate and analyze more complete changes in search trends, we first chose to download data from 2004. Although the search data are updated in real time, as mentioned above, the publication of cancer registration data usually lag behind data collection for several years; thus, the latest incidence and mortality rates of lung cancer available for the present analysis were those published in 2015 by the CDC. To explore the association of internet search volumes for lung cancer with published cancer incidence and mortality rates in the United States, we downloaded the relevant data for the same period of time and set the year 2015 as our first deadline. We downloaded data in January 2019 so that we could obtain complete search data from 2004 to 2018 before conducting the subsequent analysis.

### Search Query Terms

Lung cancer awareness was examined on the basis of the general population’s ability to seek such information or pay attention to it [33]. We selected 12 different query terms among the most commonly used terms for lung cancer [34]. The selected terms were not searched in quotes. Search query volumes were filtered by the term “health” using the Google query category feature to discard non health-related queries that may have confounded the results. Each data point represented the relative search volume (RSV) of specific query terms on a normalized scale of 0-100. The data were divided by total searches of a particular geographic location and the particular time range they represent to compare the relative popularity of the query terms. For example, compared with the total search volumes, if a particular region had a higher number of specific query terms, the RSV would be closer to 100. Data of internet searches used in this study are publicly available, anonymous, and cannot be traced back to identifiable individuals.

### Cancer Data

The age-adjusted incidence and mortality rates per 100,000 individuals with lung cancer in both sexes were obtained at the state and national levels from the CDC for the period of 2004-2015 [2]. Incidence and mortality rates were then merged by state and integrated with the monthly RSV data; the processed dataset included 144 data points for each state for a total of 7344 data points.

### Statistical Analyses

RSVs at national and state levels are represented as time-series data. Each time series was divided into four parts: long-term trend, seasonal change, cycling, and random fluctuation. We used a time-series decomposition method to eliminate the effects of irregular changes and obtained the long-term trends of the internet search data from January 2004 to December 2018. Each long-term trend is a continuously increasing or decreasing trend that an objective phenomenon exhibits over a long period of time, which may be due to a fundamental cause. The purposes of studying long-term trends are to understand the regularity of the development of internet search data, provide the necessary conditions for statistical prediction, and remove them from the time series to ultimately analyze the influence of other factors on the time series. The steps of this procedure were as follows. First, we chose a multiplication model based on the time-series graph of the RSV of each state. Second, we used a moving-average method to smooth the time series, and used a monthly average method to calculate the seasonal index. Third, we drew a scatter plot and selected the long-term trend of the appropriate curve model to fit the sequence and obtain the long-term trend. Finally, after decomposing the seasonal index and the periodic variation factors, the remaining factors represented the long-term trend.

We performed Spearman correlation analysis to evaluate the relationship between the known lung cancer incidence and mortality rates and Google RSVs over the period of 2004-2015. As Spearman correlation analysis is a nonparametric method, no assumptions of distributions were required on the original variables. We also performed Spearman correlation analysis to evaluate the relationship among the incidence and mortality rates and search data after time-series decomposition.

We assumed that trends in lung cancer-related internet hits would be consistent with those reported for national lung cancer incidence. As the search data could be downloaded in real time, we downloaded complete search data from 2004 to 2018. However, publication of incidence and mortality rates usually lags 3 years behind the actual data. Hence, after the correlation analysis, we conducted a simple linear prediction of the incidence and mortality rates of lung cancer from 2016 to 2018 based on previous epidemiological data, and explored whether trends in lung cancer internet hits would be consistent with the future incidence and mortality rates of lung cancer in the same manner as described above. Therefore, we set the year 2018 as our second deadline for the present analysis.

Statistical analysis was conducted using IBM SPSS (version 22.0, IBM Corporation, Armonk, NY, USA), EViews (version 8, IHS Global Inc, London, UK), and R project (version 3.4, R Development Core Team, Vienna, Austria). The statistical significance of the correlation for each dataset was computed using a two-tailed Student *t* test. The significance level was set at alpha=.05 for all tests.

## Results

### Burden of Lung Cancer

**Figure 1:**
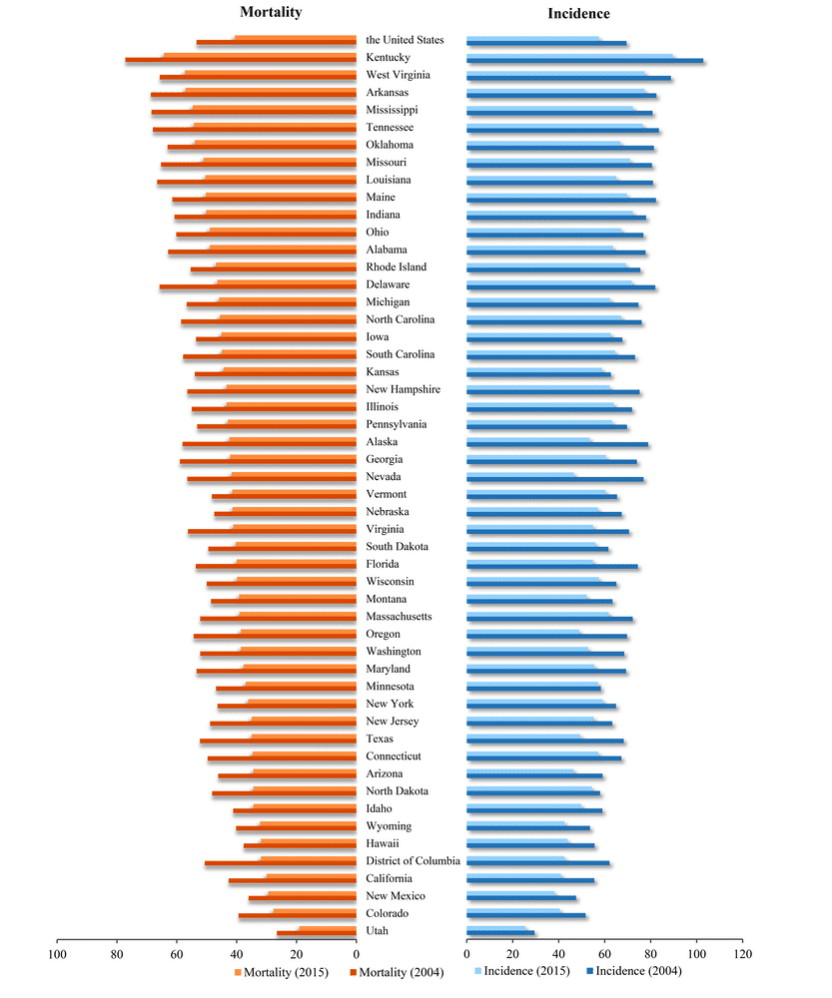
Mortality and incidence rates of lung cancer in the United States broken down by state in 2004 and 2015.

Figure 1 shows the ranking of lung cancer rates in each state in 2004 and 2015. Compared with those in 2004, both rates declined to varying degrees in all states by 2015. Kentucky showed the highest incidence and mortality rates of lung cancer, whereas Utah showed the lowest incidence and mortality rates of lung cancer.

### Inclusion of Search Query Terms

We separately extracted the Google RSVs for each of the 12 keywords selected in the above method. However, except for “lung cancer,” the other query terms lacked Google RSVs for all states. Thus, we only selected the common lay term “lung cancer” in this study in consideration of the integrity of the data (see Multimedia Appendix 1).

### Regional Distribution of Relative Search Volumes

**Figure 2:**
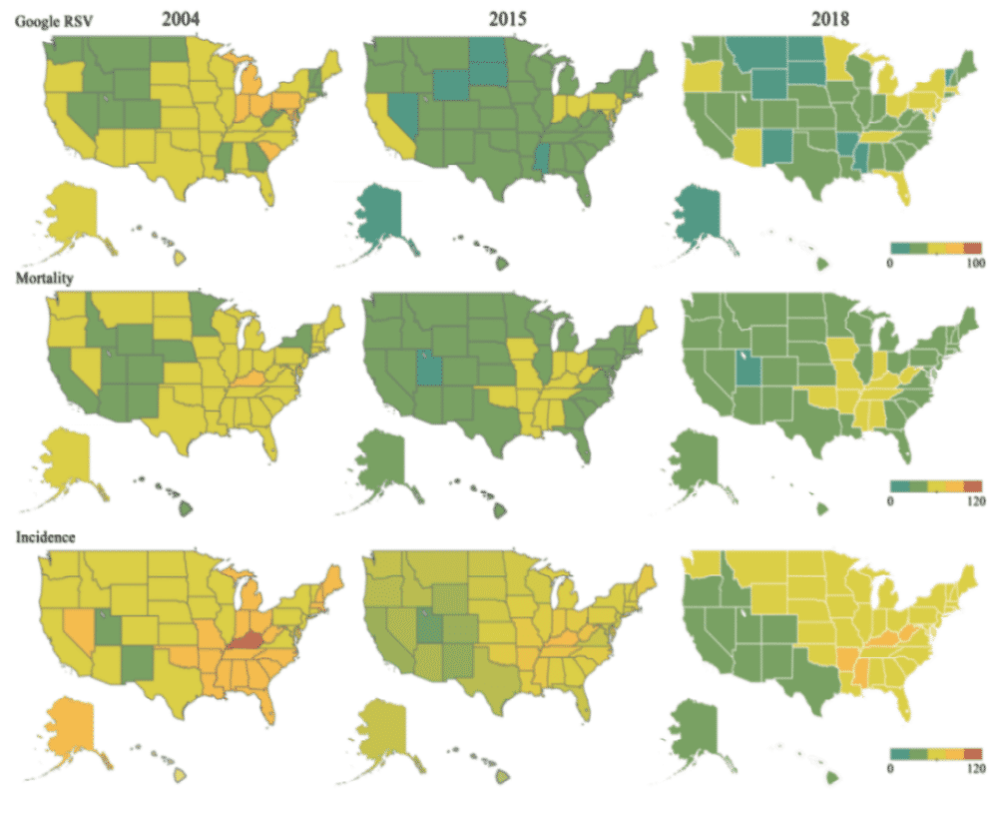
Regional distribution of mortality, incidence rates, and relative search volumes (RSVs) for lung cancer in the United States in 2004, 2015, and 2018. The color of the map changes from blue to red; the closer the color is to red, the higher the value.

Figure 2 shows the regional distributions of the Google RSVs, mortality, and incidence data for lung cancer in 2004, 2015, and 2018. The change in color from green to red in the heatmap indicates low to high values of the datasets, respectively. From 2004 to 2015, the following states had the highest incidences of lung cancer (ranked in the top 5 at any point): Kentucky, West Virginia, Tennessee, Delaware, Arkansas, Mississippi, Maine, Oklahoma, Missouri, and Indiana. The following states had the highest mortality rates (ranked in the top 5 at any point): Kentucky, Arkansas, West Virginia, Tennessee, Mississippi, Oklahoma, and Louisiana. The five states with higher average RSVs (>50 from 2004 to 2015) were as follows: Ohio, Maryland, Connecticut, Indiana, and Pennsylvania. The three states with lower average RSVs (<20) were as follows: Mississippi, Wyoming, and North Dakota. Thus, the states with higher incidence and mortality rates usually had higher RSVs (Figure 2).

### Trends in Internet Searches Related to the Incidence and Mortality Rates of Lung Cancer

**Figure 3:**
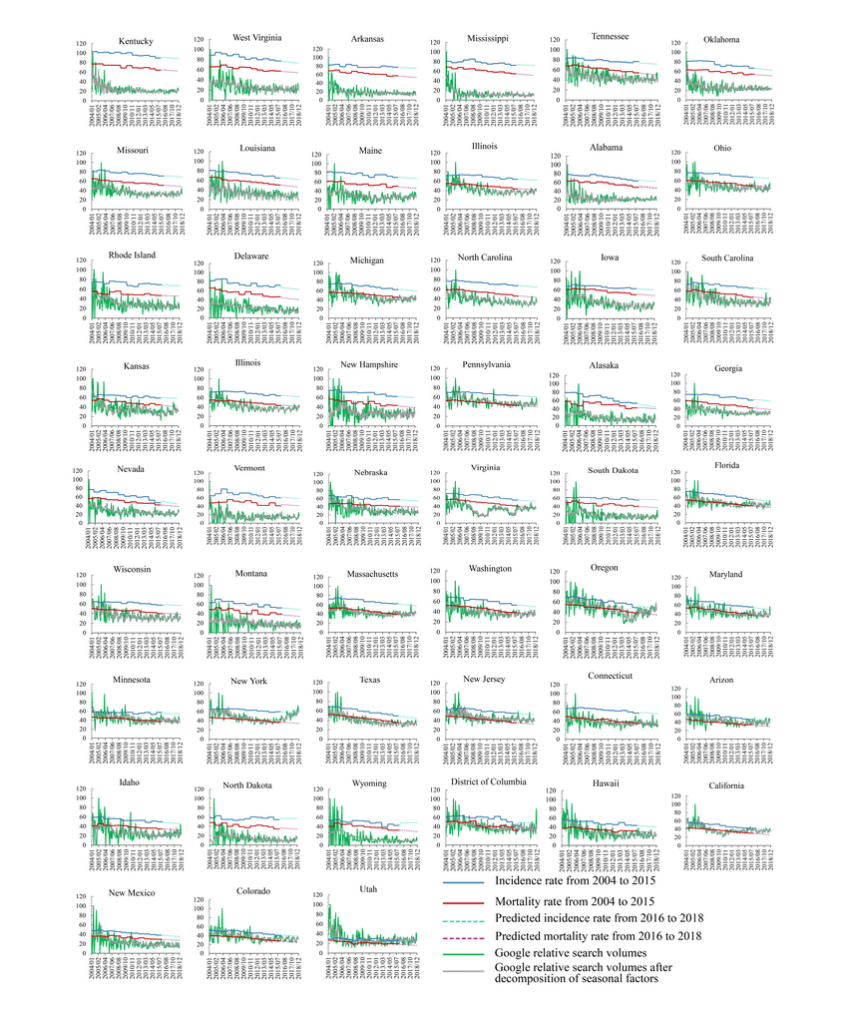
Time series of internet search data, incidence, and mortality rates from 2004 to 2018 in the United States. The vertical axis represents the rate and Google relative search volumes, and the horizontal axis represents time. The unit of rate is the number of patients or deaths per 100,000 people.

Figure 3 shows a time series of the Google RSVs, along with the incidence (blue lines) and mortality (red lines) rates of lung cancer of all states from 2004 to 2018. The green lines represent the original RSVs, the gray lines represent the new RSVs after seasonal decomposition, and the dotted lines represent the predicted value (from 2016 to 2018). The trends of RSVs for these states initially fluctuated but eventually showed a steady trend over time. The incidence and mortality rates of lung cancer in each state showed a downward trend with time.

### Correlational Analyses

**Figure 4:**
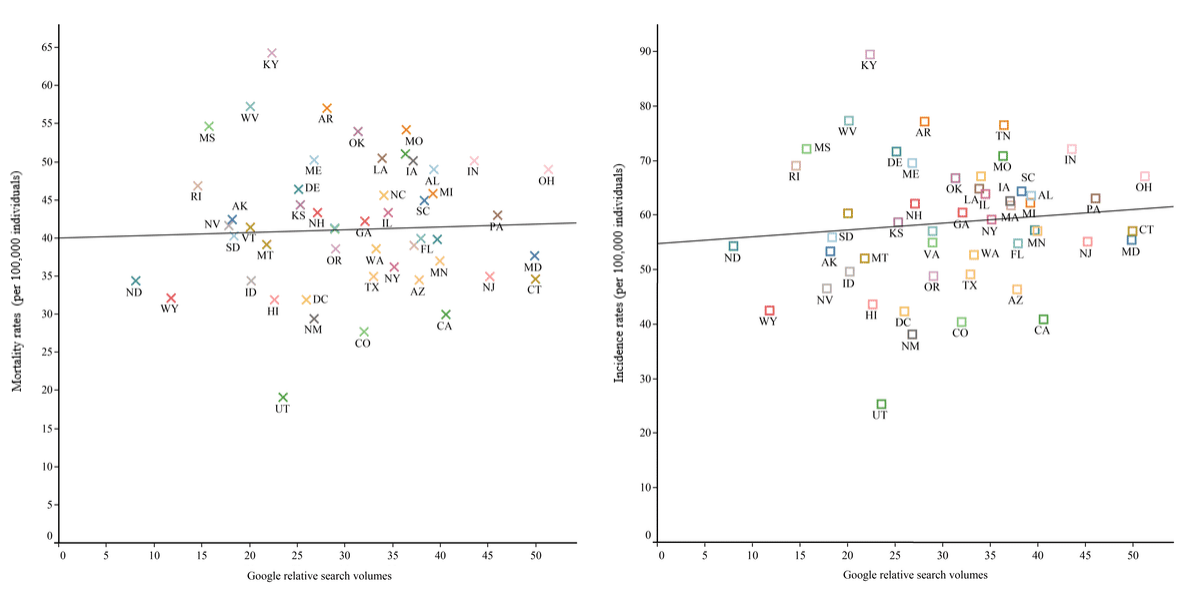
Scatter plots with fitted linear regression lines of mortality and incidence rates vs relative search volumes for lung cancer by state. Note that linear regression lines are not equivalent to correlation coefficients and are only included for visual purposes.

Multimedia Appendix 2 shows the Spearman correlation coefficients among incidence rates of lung cancer, mortality rates of lung cancer, and Google RSVs by state in the United States. We found statistically significant correlations between incidence rates and original Google RSVs for lung cancer in 42 of 51 states, with the only exceptions being Arkansas, California, Connecticut, District of Columbia, North Dakota, Rhode Island, Utah, Vermont, and West Virginia. We also found statistically significant correlations between mortality rates and Google RSVs for lung cancer for all but four states. For California, Connecticut, and Vermont, there were no statistically significant correlations between incidence rates and relative Google search volumes, and there were also no significant correlations between mortality rates and Google RSVs. Following time-series decomposition of the RSVs, both the lung cancer incidence and mortality rates were correlated with Google RSVs in 50 states, except for Vermont. Figure 4 shows a representative scatter plot of incidence rates, mortality rates, and relative Google RSVs of lung cancer by state in 2015.

## Discussion

### Principal Findings

Previous studies have mainly focused on the epidemiology of infectious diseases by analyzing internet searches or using social media data sources (eg, Twitter) to conduct health research. The present study examined the association between internet searches and the incidence and mortality rates of lung cancer at national and state levels in the United States over a 12-year period, and predicted the incidence and mortality rates of lung cancer based on their correlations with internet searches. We found an association between RSVs and actual incidence rates of lung cancer for 42 states. Upon examining lung cancer mortality rates, we found statistically significant correlations between mortality rates and RSVs for all but four states.

Our results support the concept of using internet search data and broader public access in health topics for estimating disease characteristics such as incidence and mortality rates. For Kentucky, which had the highest lung cancer incidence and mortality rates in the United States, both incidence and mortality rates were strongly correlated with Google RSVs. In Utah, which had the lowest incidence and mortality rates of lung cancer, RSVs were not correlated with either of these two rates. One possible explanation for this pattern could be that prior online search activities are amplified by individuals at risk for lung cancer, their caregivers, or those who want to acquire knowledge on lung cancer [35-37]. Our results further suggest that search behaviors may reflect, at least partially, the actual prevalence of lung cancer in each state. These types of data sources can be particularly useful when real-time information is needed, because the publication of cancer registration data usually lag several years from data collection. Previous studies have also confirmed that many data elements discernible from a user’s social media, especially demographics, can provide new opportunities to characterize the users whose data are analyzed in health research. Google and Twitter-based health research is a growing field that can be utilized to conduct content analysis, surveillance, engagement, intervention, and network analysis in health fields [38,39]. In 37 states, the correlation coefficients between mortality rates and RSVs were higher than those between incidence rates and RSVs. This situation may indicate that people pay more attention to the death caused by lung cancer, implying a general lack awareness of prevention, and that relevant departments are not paying enough attention to the prevention and intervention of lung cancer.

Many other factors may contribute to this association. Although most of the states surveyed in the present study showed statistically significant correlations of RSVs with incidence and mortality rates, some states did not show such correlations (eg, Vermont). However, our study contained only one query term, which was not able to reflect people’s search needs and changes in search patterns. Additionally, data downloaded from Google Trends are not raw and unprocessed data, and Google’s search algorithm is not static. The algorithm itself is constantly being tested and improved. The instability of Google searching caused by algorithmic dynamics may induce Google Trends to also offer an unstable depiction of the occurrence of lung cancer. Algorithmic dynamics represent changes made by engineers to improve business services and consumer use of their search services. Google’s search algorithms and user behavior changes can affect the tracking of Google Trends. The multitude of algorithmic changes is the result of Google’s numerous programmers working on small units combined with the use of search engines by millions of consumers around the world. Hence, it is difficult to reproduce the original algorithm of Google Trends. When users search for disease information, Google may automatically provide more entries that are helpful for diagnosis, such that changes in each user’s search result in changes in the Google Trends due to the recommendation of the search bar. Google does offer a function called “related queries,” which allows users to identify the retrieved data after a given time series, but this only applies to data at the national level. The key concepts are the lack of transparency regarding absolute search volumes, the exact query text and search Boolean logic used to calculate search volumes, and preprocessing in the temporal domain. After processing by Google, the filtered data may have lost much of the information contained in the original raw data [40]. Several studies have attempted to ameliorate this shortcoming by supplementing search data from other search engines such as Bing and Baidu. These search engines are capable of providing more granular search information (eg, gender, age, and regional information of the search population) than is available from Google Trends [41].

Twitter, Facebook, Google, and the entire internet are changing day by day. Studying these changes, trends, and algorithms may help us to develop more efficient data analysis models. This situation may also be influenced by various public health activities related to lung cancer. The launch of these activities may increase the public’s online searches for lung cancer, regardless of disease indicators. One study found that RSVs increased in lung cancer screening after trials reported their potential to reduce the risk of death in heavy smokers [42]. Previous studies have found that after the public health campaign for a disease has been launched, information search behavior related to the disease increases. For example, every October, the annual breast cancer campaign in the United States stimulates online search activities, and the number of Google searches for “breast cancer” has since increased significantly [43]. Moreover, RSVs for lung cancer increased after trials reported the potential of screening to reduce the risk of death among heavy smokers [44]. Reports of cancer in famous individuals may also lead to an increase in internet searches, which has been called “the celebrity effect” [30].

There are also regional differences in search volumes. In recent years, the emergence of corresponding therapy and new early screening methods for lung cancer have helped to prevent and treat lung cancer, but the level of development of lung cancer prevention and treatment, along with the diagnostic rate and survival time of lung cancer, vary from state to state. It is well known that indoor and outdoor air pollution increases the incidence of lung cancer, including smoking, air pollution, secondhand smoke, and radon [45,46]. For example, Kentucky has a high smoking rate and a relatively high incidence of lung cancer, whereas Utah has a low smoking rate and a relatively low incidence of lung cancer. Local authorities should strive to ensure that online health information is available to the public, especially in areas with high smoking rates and lung cancer incidence. Researchers and public health practitioners can further explore this situation by accessing and analyzing recent Google Trends data, which may assist with predicting regions of concern for lung cancer. Additional research on this topic may help to determine how organizations might use Google Trends data as a tool for prediction and targeted interventions. Google Trends can also be used to measure the development of knowledge and interest in new cancer screening tests or specific screening tests. Some scholars have found that people are considerably interested in lung cancer screenings and virtual colonoscopies, but are not very interested in prostate cancer screening [44]. Researchers could explore the psychology behind why people search for lung cancer, such as whether they search primarily for information related to experiencing potential symptoms, have heard of public figures suffering from lung cancer, or have read other information on the news to prompt such searches [47-49]. Additionally, as keyword predictors vary over time, additional research could investigate how searches may relate to current behavioral trends. Our study suggests that Google Trends data could be a new data source for researchers and organizations focused on addressing lung cancer. In the United States, where lung cancer is reported by the CDC and the NCI Surveillance, Epidemiology, and End Results Program through traditional epidemiological methods, supplementing case report data with extant data sources like Google Trends data may help enhance current surveillance by forecasting changes in informing targeted awareness campaigns.

We also forecasted the incidence and mortality rates of lung cancer in all states from 2016 to 2018 based on correlations between these rates and Google RSVs. Our direction for future work may focus on predicting the incidence of lung cancer by combining RSV data and real-world medical data. This approach may not only benefit the development of early warning, intervention, prevention, and control measures for lung cancer but may also extend the research findings of lung cancer to other chronic diseases.

### Limitations

Studying search engine data is inevitably restricted by some random factors. As such, the present study had some limitations. First, since the internet search data of other query terms in some states were not included in Google Trends, the present study only contained one search term. Second, Google Trends is not a real epidemiological instrument. The use of Google Trends to estimate lung cancer is not fully representative of the general population, since only individuals with access to the internet can be accounted for via this approach. Third, we were unable to determine which types of internet users conducted search activities. Fourth, we only utilized search data from one search engine, which may not represent search preferences of the whole population, although Google leads the explicit core search market in the United States [32,50]. We hope to continue to find novel ways to identify and reduce biases in search engine data for ultimately utilizing internet search data to provide useful information for cancer surveillance, evaluation of public cancer awareness, and education programs.

### Conclusions

The widespread proliferation and increasing utility of the internet have fundamental impacts on the ways in which people seek and acquire medical information. Studying internet search data can augment traditional methods for cancer surveillance when registry data are lagging, and help to achieve improved prevention and control of diseases. In addition, underscoring the potential utility of internet searches may be beneficial to identify people or regions at risk for cancers or other chronic noncommunicable diseases.

## Appendix

Multimedia Appendix 1 Selected search query terms and data available in each state.

Multimedia Appendix 2 Correlation coefficients between lung cancer rates and relative search volumes (RSVs).

## Abbreviations

CDC: US Centers for Disease Control and Prevention
NCI: National Cancer Institute
RSV: relative search volume

## References

[1] Bray F, Ferlay J, Soerjomataram I, Siegel RL, Torre LA, Jemal A (2018). Global cancer statistics 2018: GLOBOCAN estimates of incidence and mortality worldwide for 36 cancers in 185 countries. CA Cancer J Clin.

[2] (2017). United States Cancer Statistics: Data Visualizations.

[3] Siegel RL, Miller KD, Jemal A (2018). Cancer statistics, 2018. CA Cancer J Clin.

[4] Siegel RL, Miller KD, Jemal A (2019). Cancer statistics, 2019. CA Cancer J Clin.

[5] McCabe MS, Bhatia S, Oeffinger KC, Reaman GH, Tyne C, Wollins DS, Hudson MM (2013). American Society of Clinical Oncology statement: achieving high-quality cancer survivorship care. J Clin Oncol.

[6] Bove R (2019). Social Media in the Age of the. N Engl J Med.

[7] Merchant RM, Elmer S, Lurie N (2011). Integrating social media into emergency-preparedness efforts. N Engl J Med.

[8] Hartzband P, Groopman J (2010). Untangling the Web--patients, doctors, and the Internet. N Engl J Med.

[9] Lemire Marc, Paré Guy, Sicotte Claude, Harvey Charmian (2008). Determinants of Internet use as a preferred source of information on personal health. Int J Med Inform.

[10] Chen Y, Li C, Liang J, Tsai C (2018). Health Information Obtained From the Internet and Changes in Medical Decision Making: Questionnaire Development and Cross-Sectional Survey. J Med Internet Res.

[11] Leonelli S (2019). The challenges of big data biology. Elife.

[12] Munevar S (2017). Unlocking Big Data for better health. Nat Biotechnol.

[13] Simonsen L, Gog JR, Olson D, Viboud C (2016). Infectious Disease Surveillance in the Big Data Era: Towards Faster and Locally Relevant Systems. J Infect Dis.

[14] Miniwatts MG World internet users statistics and 2019 world population stats.

[15] Kepios Pte (2019). Digital 2019: Global Digital Overview.

[16] The World Bank http://wdi.worldbank.org/table/5.12.

[17] Pew Research Center Mobile Fact Sheet, February 2018.

[18] Salathé Marcel (2016). Digital Pharmacovigilance and Disease Surveillance: Combining Traditional and Big-Data Systems for Better Public Health. J Infect Dis.

[19] Bidargaddi N, Musiat P, Makinen V, Ermes M, Schrader G, Licinio J (2017). Digital footprints: facilitating large-scale environmental psychiatric research in naturalistic settings through data from everyday technologies. Mol Psychiatry.

[20] Sinnenberg L, Buttenheim AM, Padrez K, Mancheno C, Ungar L, Merchant RM (2017). Twitter as a Tool for Health Research: A Systematic Review. Am J Public Health.

[21] Okon E, Rachakonda V, Hong HJ, Callison-Burch C, Lipoff JB (2019). Natural language processing of Reddit data to evaluate dermatology patient experiences and therapeutics. J Am Acad Dermatol.

[22] Zhan Y, Liu R, Li Q, Leischow SJ, Zeng DD (2017). Identifying Topics for E-Cigarette User-Generated Contents: A Case Study From Multiple Social Media Platforms. J Med Internet Res.

[23] Google Google Trends.

[24] Young SD, Torrone EA, Urata J, Aral SO (2018). Using Search Engine Data as a Tool to Predict Syphilis. Epidemiology.

[25] Deiner MS, Lietman TM, McLeod SD, Chodosh J, Porco TC (2016). Surveillance Tools Emerging From Search Engines and Social Media Data for Determining Eye Disease Patterns. JAMA Ophthalmol.

[26] Santillana M (2017). Editorial Commentary: Perspectives on the Future of Internet Search Engines and Biosurveillance Systems. Clin Infect Dis.

[27] Ginsberg J, Mohebbi MH, Patel RS, Brammer L, Smolinski MS, Brilliant L (2009). Detecting influenza epidemics using search engine query data. Nature.

[28] Yang S, Santillana M, Kou SC (2015). Accurate estimation of influenza epidemics using Google search data via ARGO. Proc Natl Acad Sci U S A.

[29] Lu FS, Hattab MW, Clemente CL, Biggerstaff M, Santillana M (2019). Improved state-level influenza nowcasting in the United States leveraging Internet-based data and network approaches. Nat Commun.

[30] Wehner MR, Nead KT, Linos E (2017). Correlation Among Cancer Incidence and Mortality Rates and Internet Searches in the United States. JAMA Dermatol.

[31] Phillips CA, Barz Leahy A, Li Y, Schapira MM, Bailey LC, Merchant RM (2018). Relationship Between State-Level Google Online Search Volume and Cancer Incidence in the United States: Retrospective Study. J Med Internet Res.

[32] Statcounter (2019). Search engine market share worldwide.

[33] Ji Z, Zhang Y, Xu J, Chen X, Wu Y, Xu H (2017). Comparing Cancer Information Needs for Consumers in the US and China. Stud Health Technol Inform.

[34] Merck Manual Professional Edition, Online edition Lung Carcinoma: Tumors of the Lungs.

[35] Arora NK, Hesse BW, Rimer BK, Viswanath K, Clayman ML, Croyle RT (2008). Frustrated and confused: the American public rates its cancer-related information-seeking experiences. J Gen Intern Med.

[36] Kowalski C, Kahana E, Kuhr K, Ansmann L, Pfaff H (2014). Changes over time in the utilization of disease-related Internet information in newly diagnosed breast cancer patients 2007 to 2013. J Med Internet Res.

[37] Castleton K, Fong T, Wang-Gillam A, Waqar MA, Jeffe DB, Kehlenbrink L, Gao F, Govindan R (2011). A survey of Internet utilization among patients with cancer. Support Care Cancer.

[38] Huang X, Baade P, Youlden DR, Youl PH, Hu W, Kimlin MG (2017). Google as a cancer control tool in Queensland. BMC Cancer.

[39] Pershad Y, Hangge P, Albadawi H, Oklu R (2018). Social Medicine: Twitter in Healthcare. J Clin Med.

[40] Google How Trends data is adjusted.

[41] Xu C, Wang Y, Yang H, Hou J, Sun L, Zhang X, Cao X, Hou Y, Wang L, Cai Q, Wang Y (2019). Association Between Cancer Incidence and Mortality in Web-Based Data in China: Infodemiology Study. J Med Internet Res.

[42] McCook A More signs lung cancer screening could save lives, 2010.

[43] Glynn RW, Kelly JC, Coffey N, Sweeney KJ, Kerin MJ (2011). The effect of breast cancer awareness month on internet search activity--a comparison with awareness campaigns for lung and prostate cancer. BMC Cancer.

[44] Schootman M, Toor A, Cavazos-Rehg P, Jeffe DB, McQueen A, Eberth J, Davidson NO (2015). The utility of Google Trends data to examine interest in cancer screening. BMJ Open.

[45] Simon S American Cancer Society.

[46] Alberg AJ, Brock MV, Ford JG, Samet JM, Spivack SD (2013). Epidemiology of lung cancer: Diagnosis and management of lung cancer, 3rd ed: American College of Chest Physicians evidence-based clinical practice guidelines. Chest.

[47] Ellery PJ, Vaughn W, Ellery J, Bott J, Ritchey K, Byers L (2013). Understanding internet health search patterns: An early exploration into the usefulness of Google Trends. J Commun Healthc.

[48] McMullan M (2006). Patients using the Internet to obtain health information: how this affects the patient-health professional relationship. Patient Educ Couns.

[49] Steehler KR, Steehler MK, Pierce ML, Harley EH (2013). Social media's role in otolaryngology-head and neck surgery: informing clinicians, empowering patients. Otolaryngol Head Neck Surg.

[50] Comscore Releases February 2016.

