# Supplementary material for: Detecting Lung Cancer Trends by Leveraging Real-World and Internet-Based Data: Infodemiology Study
Source: J Med Internet Res. 2020 Mar 12;22(3):e16184. doi: 10.2196/16184 (PMC7099398; doi:10.2196/16184)
Supplement: Multimedia Appendix 1 [file jmir_v22i3e16184_app1.doc]

| **Multimedia appendix 1:** Selected search query terms and data available in each state. | | | | | | | | | | | | |
| --- | --- | --- | --- | --- | --- | --- | --- | --- | --- | --- | --- | --- |
| state | 1 | 2 | 3 | 4 | 5 | 6 | 7 | 8 | 9 | 10 | 11 | 12 |
|  | lung cancer | lung carcinoma | tumors of the lungs | small cell lung cancer | small cell lung carcinoma | SCLC | non small cell lung cancer | non-small cell lung cancer | NSCLC | non-small cell lung carcinoma | non small cell lung carcinoma | carcinoma of the lungs |
| Alabama | √ | √ | √ | √ |  | √ | √ |  | √ |  |  |  |
| Alaska | √ |  |  |  |  |  |  |  |  |  |  |  |
| Arizona | √ | √ | √ |  |  | √ | √ |  | √ |  |  |  |
| Arkansas | √ |  |  | √ |  |  |  |  |  |  |  |  |
| California | √ | √ |  | √ |  | √ | √ | √ | √ |  |  |  |
| Colorado | √ |  |  | √ |  | √ |  |  | √ |  |  |  |
| Connecticut | √ |  |  | √ |  | √ | √ |  | √ |  |  |  |
| Delaware | √ |  |  |  |  |  |  |  |  |  |  |  |
| District of Columbia | √ |  |  |  |  | √ |  |  | √ |  |  |  |
| Florida | √ | √ |  | √ | √ | √ | √ |  | √ |  |  |  |
| Georgia | √ | √ |  | √ |  | √ | √ |  | √ |  |  |  |
| Hawaii | √ |  |  |  |  | √ |  |  |  |  |  |  |
| Idaho | √ |  |  |  |  | √ |  |  |  |  |  |  |
| Illinois | √ | √ |  | √ |  | √ | √ |  | √ |  |  |  |
| Indiana | √ | √ |  | √ |  | √ | √ |  | √ |  |  |  |
| Iowa | √ |  |  | √ |  | √ |  |  |  |  |  |  |
| Kansas | √ |  |  | √ |  | √ |  |  | √ |  |  |  |
| Kentucky | √ | √ |  | √ |  | √ | √ |  | √ |  |  |  |
| Louisiana | √ | √ |  | √ |  | √ |  |  | √ |  |  |  |
| Maine | √ |  |  | √ |  | √ |  |  |  |  |  |  |
|  |  |  |  |  |  |  |  |  |  |  |  |  |
| Maryland | √ | √ |  |  |  | √ | √ |  | √ |  |  |  |
| Massachusetts | √ | √ |  | √ |  | √ | √ |  | √ |  |  |  |
| Michigan | √ | √ |  | √ |  | √ | √ |  | √ |  |  |  |
| Minnesota | √ | √ |  | √ |  | √ |  |  | √ |  |  |  |
| Mississippi | √ |  |  | √ |  | √ |  |  |  |  |  |  |
| Missouri | √ | √ |  | √ |  | √ | √ |  | √ |  |  |  |
| Montana | √ |  |  |  |  | √ |  |  |  |  |  |  |
| Nebraska | √ |  |  | √ |  | √ |  |  |  |  |  |  |
| Nevada | √ |  |  | √ |  | √ |  |  |  |  |  |  |
| New Hampshire | √ |  |  |  |  | √ |  |  |  |  |  |  |
| New Jersey | √ | √ |  | √ |  | √ | √ |  | √ |  |  |  |
| New Mexico | √ |  |  |  |  | √ |  |  |  |  |  |  |
| New York | √ | √ |  | √ | √ | √ | √ |  | √ |  | √ |  |
| North Carolina | √ | √ |  | √ |  | √ | √ | √ | √ |  |  |  |
| North Dakota | √ |  |  |  |  | √ |  |  |  |  |  |  |
| Ohio | √ | √ |  | √ |  | √ | √ |  | √ |  |  |  |
| Oklahoma | √ |  |  | √ |  | √ |  |  | √ |  |  |  |
| Oregon | √ |  |  | √ |  | √ |  |  | √ |  |  |  |
| Pennsylvania | √ |  |  | √ |  | √ | √ |  | √ |  |  |  |
| Rhode Island | √ |  |  | √ |  | √ |  |  |  |  |  |  |
| South Carolina | √ |  |  | √ |  | √ | √ |  | √ |  |  |  |
| South Dakota | √ |  |  |  |  | √ |  |  |  |  |  |  |
| Tennessee | √ | √ |  | √ |  | √ | √ |  | √ |  |  |  |
|  |  |  |  |  |  |  |  |  |  |  |  |  |
|  |  |  |  |  |  |  |  |  |  |  |  |  |
|  |  |  |  |  |  |  |  |  |  |  |  |  |
| Texas | √ | √ |  | √ | √ | √ | √ |  | √ |  |  |  |
| Utah | √ |  |  |  |  | √ |  |  |  |  |  |  |
| Vermont | √ |  |  |  |  |  |  |  |  |  |  |  |
| Virginia | √ | √ |  | √ |  | √ | √ |  | √ |  |  |  |
| Washington | √ | √ |  | √ |  | √ | √ |  | √ |  |  |  |
| West Virginia | √ |  |  | √ |  | √ |  |  |  |  |  |  |
| Wisconsin | √ | √ |  | √ |  | √ |  |  | √ |  |  |  |
| Wyoming | √ |  |  |  |  |  |  |  |  |  |  |  |
