# Supplementary material for: Detecting Lung Cancer Trends by Leveraging Real-World and Internet-Based Data: Infodemiology Study
Source: J Med Internet Res. 2020 Mar 12;22(3):e16184. doi: 10.2196/16184 (PMC7099398; doi:10.2196/16184)
Supplement: Multimedia Appendix 2 [file jmir_v22i3e16184_app2.docx]

|  | | | | | | | | | | | | |
| --- | --- | --- | --- | --- | --- | --- | --- | --- | --- | --- | --- | --- |
|  | **Ranking of mortality**  **rates** | | **Ranking of incidence rates** | | **Correlation between incidence and original**  **RSV** | | **Correlation between mortality and original RSV** | | **Correlation between incidence and smoothed RSV** | | **Correlation between**  **mortality and smoothed RSV** | |
| **State** | **2004** | **2015** | **2004** | **2015** | **R** | ***P* Value** | **R** | ***P* Value** | **R** | ***P* Value** | **R** | ***P* Value** |
| the United States | - | - | - | - | 0.755 | <.001 | 0.408 | <.001 | 0.877 | <.001 | 0.394 | <.001 |
| Kentucky | 1 | 1 | 1 | 1 | 0.824 | .001 | 0.781 | .003 | 0.501 | <.001 | 0.555 | <.001 |
| West Virginia | 7 | 2 | 2 | 2 | 0.496 | .10 | 0.740 | .006 | 0.734 | <.001 | 0.688 | <001 |
| Arkansas | 2 | 3 | 4 | 3 | 0.421 | .17 | 0.678 | .02 | 0.356 | <.001 | 0.604 | <.001 |
| Mississippi | 3 | 4 | 9 | 5 | 0.705 | .01 | 0.832 | .001 | 0.21 | .01 | 0.316 | <.001 |
| Tennessee | 4 | 5 | 3 | 4 | 0.849 | <.001 | 0.910 | <.001 | 0.568 | <.001 | 0.639 | <.001 |
| Oklahoma | 9 | 6 | 7 | 13 | 0.621 | .03 | 0.575 | .05 | 0.323 | <.001 | 0.32 | <.001 |
| Missouri | 8 | 7 | 10 | 8 | 0.868 | <.001 | 0.868 | <.001 | 0.791 | <.001 | 0.816 | <.001 |
| Louisiana | 5 | 8 | 8 | 14 | 0.858 | <.001 | 0.882 | <.001 | 0.605 | <.001 | 0.609 | <.001 |
| Maine | 11 | 9 | 5 | 9 | 0.593 | .04 | 0.595 | .04 | 0.651 | <.001 | 0.683 | <.001 |
| Indiana | 12 | 10 | 12 | 6 | 0.673 | .02 | 0.772 | .003 | 0.632 | <.001 | 0.651 | <.001 |
| Alabama | 10 | 11 | 13 | 17 | 0.742 | .006 | 0.815 | .001 | 0.214 | <.001 | 0.283 | .001 |
| Ohio | 13 | 12 | 15 | 12 | 0.773 | .003 | 0.775 | .003 | 0.714 | <.001 | 0.745 | <.001 |
| Rhode Island | 22 | 13 | 17 | 10 | 0.517 | .09 | 0.650 | .02 | 0.481 | <.001 | 0.609 | <.001 |
| Delaware | 6 | 14 | 6 | 7 | 0.753 | .005 | 0.781 | .003 | 0.763 | <.001 | 0.795 | <.001 |
| Michigan | 18 | 15 | 19 | 20 | 0.865 | <.001 | 0.881 | <.001 | 0.785 | <.001 | 0.818 | <.001 |
| North Carolina | 15 | 16 | 16 | 11 | 0.907 | <.001 | 0.942 | <.001 | 0.843 | <.001 | 0.86 | <.001 |
| Iowa | 27 | 17 | 31 | 19 | 0.867 | <.001 | 0.824 | .001 | 0.737 | <.001 | 0.766 | <.001 |
| South Carolina | 17 | 18 | 22 | 15 | 0.891 | <.001 | 0.905 | <.001 | 0.713 | <.001 | 0.785 | <.001 |
| Kansas | 25 | 19 | 39 | 26 | 0.667 | .02 | 0.902 | <0.001 | 0.238 | .004 | 0.446 | <.001 |
| Illinois | 23 | 20 | 24 | 16 | 0.782 | .003 | 0.863 | <.001 | 0.736 | <.001 | 0.811 | <.001 |
| New Hampshire | 20 | 21 | 18 | 21 | 0.598 | .04 | 0.482 | .11 | 0.524 | <.001 | 0.519 | <.001 |
| Pennsylvania | 29 | 22 | 27 | 18 | 0.851 | <.001 | 0.893 | <.001 | 0.732 | <.001 | 0.763 | <.001 |
| Alaska | 16 | 23 | 11 | 37 | 0.878 | <.001 | 0.596 | .04 | 0.724 | <.001 | 0.514 | <.001 |
| Georgia | 14 | 24 | 21 | 23 | 0.696 | .01 | 0.735 | .006 | 0.8 | <.001 | 0.855 | <.001 |
| Nevada | 19 | 25 | 14 | 43 | 0.791 | .002 | 0.808 | .001 | 0.734 | <.001 | 0.752 | <.001 |
| Vermont | 39 | 26 | 34 | 24 | 0.357 | .26 | 0.104 | .75 | -0.077 | .36 | –0.078 | .36 |
| Nebraska | 41 | 27 | 32 | 30 | 0.691 | .01 | 0.762 | .004 | 0.637 | <.001 | 0.686 | <.001 |
| Virginia | 21 | 28 | 25 | 34 | 0.578 | .05 | 0.655 | .02 | 0.574 | <.001 | 0.637 | <.001 |
| South Dakota | 36 | 29 | 41 | 31 | 0.746 | .005 | 0.794 | .002 | 0.556 | <.001 | 0.631 | <.001 |
| Florida | 26 | 30 | 20 | 35 | 0.908 | <.001 | 0.894 | <.001 | 0.803 | <.001 | 0.806 | <.001 |
| Wisconsin | 34 | 31 | 35 | 27 | 0.708 | .01 | 0.828 | .001 | 0.579 | <.001 | 0.656 | <.001 |
| Montana | 38 | 32 | 37 | 39 | 0.871 | <.001 | 0.693 | .01 | 0.52 | <.001 | 0.455 | <.001 |
| Massachusetts | 32 | 33 | 23 | 22 | 0.757 | .004 | 0.778 | .003 | 0.776 | <.001 | 0.799 | <.001 |
| Washington | 31 | 34 | 29 | 38 | 0.810 | .001 | 0.871 | <.001 | 0.716 | <.001 | 0.743 | <.001 |
| Oregon | 24 | 35 | 26 | 42 | 0.943 | <.001 | 0.911 | <.001 | 0.836 | <.001 | 0.857 | <.001 |
| Maryland | 28 | 36 | 28 | 32 | 0.905 | <.001 | 0.926 | <.001 | 0.871 | <.001 | 0.863 | <.001 |
| Minnesota | 42 | 37 | 44 | 29 | 0.642 | .02 | 0.747 | .005 | 0.561 | <.001 | 0.767 | <.001 |
| New York | 43 | 38 | 36 | 25 | 0.774 | .003 | 0.905 | <.001 | 0.722 | <.001 | 0.825 | <.001 |
| Texas | 30 | 39 | 30 | 41 | 0.930 | <.001 | 0.917 | <.001 | 0.86 | <.001 | 0.867 | <.001 |
| New Jersey | 37 | 40 | 38 | 33 | 0.82 | .001 | 0.834 | .001 | 0.56 | <.001 | 0.586 | <.001 |
| Connecticut | 35 | 41 | 33 | 28 | 0.462 | .13 | 0.523 | .08 | 0.758 | <.001 | 0.722 | <.001 |
| Arizona | 44 | 42 | 42 | 44 | 0.76 | .004 | 0.845 | .001 | 0.735 | <.001 | 0.805 | <.001 |
| North Dakota | 40 | 44 | 45 | 36 | -0.099 | .76 | 0.631 | .03 | -0.197 | .02 | 0.197 | <.001 |
| Wyoming | 47 | 45 | 48 | 46 | 0.895 | <.001 | 0.790 | .002 | 0.808 | <.001 | 0.9 | <.001 |
| District of Columbia | 33 | 46 | 40 | 47 | 0.536 | .07 | 0.624 | .03 | 0.493 | <.001 | 0.563 | <.001 |
| Hawaii | 49 | 47 | 46 | 45 | 0.782 | .003 | 0.801 | .002 | 0.748 | <.001 | 0.83 | <.001 |
| California | 45 | 48 | 47 | 48 | 0.495 | .10 | 0.523 | .08 | 0.574 | <.001 | 0.588 | <.001 |
| New Mexico | 50 | 49 | 50 | 50 | 0.754 | .005 | 0.656 | .02 | 0.801 | <.001 | 0.639 | <.001 |
| Colorado | 48 | 50 | 49 | 49 | 0.678 | .02 | 0.812 | .001 | 0.713 | <.001 | 0.802 | <.001 |
| Utah | 51 | 51 | 51 | 51 | 0.194 | .55 | 0.640 | .03 | 0.244 | .003 | 0.451 | <.001 |
